# Cannabis Use and Analgesic Prescribing in UK Primary Care: A Retrospective Cohort Study of Patients with Osteoarthritis

**DOI:** 10.3390/medicines12040027

**Published:** 2025-11-10

**Authors:** Simon Erridge, Joht Singh Chandan, Krishna M. Gokhale, Christian Billinghurst, Mikael H. Sodergren

**Affiliations:** 1 Medical Cannabis Research Group, Department of Surgery and Cancer, Imperial College London, London W12 0HS, UK; 2 Curaleaf International, London W1W 7NY, UK; 3 Department of Applied Health Sciences, University of Birmingham, Birmingham B15 2TT, UK; 4 National Institute for Health and Care Research (NIHR) Birmingham Biomedical Research Centre, Birmingham B15 2TH, UK; 5 Birmingham Health Partners, Solihull B90 4AA, UK; 6 Dexter Software, 97 Vincent Drive, Birmingham B15 2SQ, UK

**Keywords:** cannabis, osteoarthritis, primary care, analgesia, opioids, chronic pain

## Abstract

Objectives: This study aims to assess differences in analgesia prescribing in UK primary care between individuals with osteoarthritis who have a recorded exposure to cannabis use and those who do not. Methods: This population-based retrospective cohort study included opioid-naïve patients with osteoarthritis (aged 25–85 years) who were active in Clinical Practice Research Datalink Aurum between 1 January 1995 and 15 December 2023. Patients with osteoarthritis who had current or historic cannabis use recorded were matched to two unexposed individuals by age, sex, smoking status, and health authority. Patients were followed up to assess prescriptions of analgesia. Cox regression was performed adjusted for age, sex, and ethnicity. Results: 662 exposed patients were matched to 1319 unexposed patients. Cannabis-exposed individuals were more likely to be prescribed opioids (adjusted hazard ratio (HR): 2.06; 95% confidence interval (CI): 1.74–2.43; *p* < 0.001), gabapentinoids (HR: 3.31; 95% CI: 2.34–4.67; *p* < 0.001), non-steroidal anti-inflammatory drugs (HR: 1.99; 95% CI: 1.72–2.31; *p* < 0.001), tricyclic antidepressants (HR: 2.64; 95% CI: 2.03–3.44; *p* < 0.001), other antidepressants (HR: 7.22; 95% CI: 5.24–9.94; *p* < 0.001), and paracetamol (HR: 3.30; 95% CI: 2.43–4.48; *p* < 0.001). Conclusions: This study suggests there is an association between coded exposure to cannabis in UK primary care records and increased prescribing of analgesia. Given the relative scarcity of recorded cannabis use relative to its prevalence in the general population, these findings must be interpreted cautiously. The increased hazard of using analgesia and mortality within the cannabis-exposed cohort may be confounded by socioeconomic status and a higher likelihood of coding cannabis use in those experiencing adverse effects after consumption or cannabis misuse disorder.

## 1. Introduction

Osteoarthritis is estimated to affect 1 in 10 individuals in the United Kingdom [1]. The condition is characterised by an imbalance between synthesis and breakdown of articular cartilage and subchondral bone, in addition to synovial inflammation, leading to loss of cartilage, sclerosis of subchondral bone, and localised joint inflammation [2]. This manifests clinically as pain, stiffness, and reduced mobility in the affected joint [2]. The chronic pain and reduction in mobility secondary to osteoarthritis are associated with reductions in physical, psychological, economic, and social health and well-being [2].

Patients with hip or knee osteoarthritis are at increased risk of all-cause mortality compared to healthy controls, in addition to cardiovascular and psychiatric comorbidities [3,4]. The direct healthcare costs of osteoarthritis in Western countries can be as high as 1–2.5% of gross domestic product, with indirect costs estimated to be in the magnitude of billions (£GBP) [5]. The prevalence of osteoarthritis in the UK is increasing at a rate of 1.4% every year according to data from the Clinical Practice Research Datalink (CPRD) [1]. Globally, the prevalence is estimated to be rising even quicker [6].

Management of osteoarthritis requires a multimodal approach. Exercise for knee osteoarthritis has high-quality evidence to support its use in improving pain and physical function when delivered in structured programmes [7,8]. Psychological therapies, in particular cognitive behavioural therapy, are associated with improvements in reducing pain [9]. However, currently available pharmacotherapeutic options are limited and moderately effective at best. Only non-steroidal anti-inflammatory drugs (NSAIDs) and intra-articular corticosteroids are supported by UK and international guidelines on non-surgical management of osteoarthritis [10,11]. Intra-articular corticosteroids can only be considered for short-term relief [10,11], and there is growing evidence to suggest that long-term administration of NSAIDs is associated with an increased risk of gastrointestinal, cardiovascular, and renal adverse events [12].

Notably, it is recommended that opioids and anti-epileptic drugs are not used in individuals with osteoarthritis-associated chronic pain [10,11]. This recommendation is based on the paucity of data to suggest they are efficacious for chronic pain, in addition to growing evidence of associated issues of dependence and other adverse events [10,11]. Yet, opioids remain the most prescribed class of analgesia for individuals with knee osteoarthritis [13]. There are no disease-modifying therapies that have been identified to date in the management of osteoarthritis.

Considering the limitations of currently available therapeutic options, it is unsurprising that there has been an increase in the number of analgesic medications prescribed per patient with osteoarthritis [13]. Moreover, the quantity of opioids administered to patients with knee osteoarthritis increased between 2000 and 2014 [13]. In 2000, 27.5 opioid prescriptions were written per 1000 patients captured in CPRD, rising to 46.5 per 1000 patients in 2014. This was also accompanied by an increase in oral morphine equivalent dose from 32.6 mg/day to 71.7 mg/day across the same period [13]. In this context, patients may seek treatments outside of traditional healthcare settings. It is estimated that 1.8 million individuals source illegal cannabis to treat diagnosed health conditions in the UK [14]. For chronic pain specifically, it was estimated that 453,255 individuals use cannabis to relieve related symptoms or side effects of a prescribed treatment [14].

The cannabis plant is thought to contain more than 540 active pharmaceutical ingredients, however the most abundant compounds are cannabinoids, particularly cannabidiol (CBD) and (-)-trans-Δ^9^-tetrahydrocannabinol (THC) [15]. The main mechanisms of action of these compounds are via the endocannabinoid system, whereby CBD inhibits the hydrolysis of endogenous cannabinoid receptor 1 (CB1) agonist, anandamide, and THC is a direct partial agonist of the CB1 receptor [16]. These cannabinoids also have additional effects on other receptors and channels which are important in neurotransmission [16].

Through these mechanisms, THC and CBD play multifactorial roles in the transmission of nociceptive stimuli, but also the modification and interpretation of pain [17]. Within peripheral sensory neurons and the dorsal root ganglia, agonism of cannabinoid receptor 2 (CB2) receptors and dose-dependent blockade of transient receptor potential vanilloid 1 (TRPV1) channels inhibits ascending nociceptive signals [18,19]. At the level of the spinal cord and brain stem, agonism of CB2 receptors helps to prevent neuronal sensitisation and the generation of negative feedback loops which are often present in individuals with chronic pain [17]. Moreover, activation of CB1 receptors activates descending inhibitory pathways within the spinothalamic tracts to further reduce the intensity of nociceptive signals reaching the brain [17].

The current clinical evidence on the effects of cannabis on chronic pain is typically of low-quality and is highly heterogeneous [20,21,22]. There have been only two randomised controlled trials (RCTs) on medical cannabis for osteoarthritis to date [23,24]. In the most recent study, 86 participants taking 3000 mg oral paracetamol per day were randomised to 600 mg oral CBD per day or placebo. At 8 weeks, there was no difference in pain between the two arms [24]. The other RCT was a phase 2A trial of synthetic CBD transdermal gel [23]. In this study, 250 mg of CBD delivered topically each day to a knee affected by osteoarthritis was associated with a 52.7% response rate compared to 34.1% in the placebo arm [23]. Considering the paucity of evidence, there are no licensed preparations of cannabis-based medicinal products (CBMPs) for osteoarthritis-associated chronic pain. Moreover, licensed CBMPs are only available via the National Health Service for treatment-resistant epilepsy, multiple sclerosis-associated spasticity, and chemotherapy-induced nausea and vomiting [25]. People who want to use cannabis for osteoarthritis-associated chronic pain in the UK therefore must access unlicensed medical cannabis preparations via private prescriptions or rely upon self-medicating via illegal channels [14,25,26].

Despite the limitations of current clinical literature on CBMPs, the current evidence suggests that CBMPs are associated with fewer adverse events when compared to opioids [27,28]. Considering the global increase in harmful opioid use and the development of opioid use disorders [29], researchers have sought to examine the effects of cannabis in reducing prescribed opioid medications. A meta-analysis including RCTs and observational studies concluded that the current evidence for the opioid-sparing effects of medical cannabis for chronic pain remain uncertain [30]. In addition, epidemiological studies from North America examining the impact of cannabis legalisation have shown mixed outcomes with respect to changes in prescriptions of opioids [31,32].

Considering the mixed outcomes to date from the opioid-sparing effects of cannabis legalisation in North America and the differences in cannabis legalisation and access to the United Kingdom, it is important to explore whether there is any effect of cannabis use on analgesia prescribing in UK patients. Yet, no studies to date have sought to examine this relationship in a UK setting. This study therefore aims to address the paucity of literature which has examined the effects of exposure to cannabis on osteoarthritis-associated chronic pain using real-world data from UK primary care. The primary aim of this study is to compare opioid prescribing between individuals with osteoarthritis and recorded exposure to cannabis against matched controls without recorded exposure to cannabis. The secondary aim is to compare prescribing of other forms of analgesia between each cohort.

## 2. Materials and Methods

### 2.1. Study Design and Data Source

A population-based retrospective cohort study was conducted utilising data from the CPRD Aurum database between 1 January 1995 and 15 December 2023. CPRD Aurum collects data from general practices in England who use EMIS Web^®^ (EMIS Health, Leeds, UK) as an electronic healthcare system [33]. It represents 46,599,092 patients in England, including those who moved to practices which do not contribute data to CPRD Aurum or died during the studied period. This data is derived from 1771 practices in England. As of December 2023, CPRD Aurum contained 16,011,762 patients who were actively registered at 1589 practices in England. This corresponds to 19.77% of UK general practices and 23.89% of the total UK population. It has been demonstrated as representative of the English population according to age, gender, geography, and levels of deprivation [33]. The data for this analysis was extracted and transformed using the Data Extraction for Epidemiological Research (DExtER) tool [34].

This study used anonymised electronic health record data from CPRD Aurum under the appropriate data sharing agreements and approvals. The study protocol was approved through the CPRD Research Data Governance process (protocol code #24_003783).

### 2.2. Study Population

The study population included all individuals with a recorded diagnosis of osteoarthritis identified using a predefined code list (Supplementary Data File S1). To be eligible, patients needed to be between 25 and 85 years at the time of cohort entry and registered with their general practitioner for a minimum of 1 year. Participants were excluded if they had a recorded prescription for one or more opioid medications prior to the index date. Participant data was censored at the end of follow-up, which was determined by patient death, change of general practice, upon reaching 115 years of age, or the final recorded appointment prior to data extraction.

### 2.3. Exposure Definition

The exposure of interest was recorded cannabis consumption, identified using a predefined clinical code list for cannabis use. The presence of the clinical code at any point during the study period or present at index was considered sufficient to include patients in the cannabis-exposed cohort. Code lists for the identification of individuals with a record of cannabis exposure can be found in Supplementary Data File S2.

### 2.4. Control Selection and Matching

Each exposed patient was matched with two unexposed controls who did not have a reported clinical code corresponding to cannabis exposure. Controls were matched by age, sex, smoking status, and health authority region at index date.

When age matching was performed, a 1-year variance was permitted between each group. For matching by smoking status, for individuals recorded as a current smoker in the cannabis exposed cohort, these were attempted to be matched to individuals recorded as being current smokers within 3 years preceding or after the exposed participant’s event date. In England, general practices have a financial incentive to record smoking status in the patients they care for [35]. Between 2006/2007 and 2013/2014, smoking status was required to be reported every 27 months. After 2013/2014, this was shortened to every 2 years [35]. Matching across a 3-year period was therefore used to ensure capture of smoking status, whilst attempting to minimise misclassification, across the study period.

### 2.5. Variables and Outcomes

In addition to variables utilised in matching cohorts, the following additional variables were extracted from the data: ethnicity, body mass index (BMI; kg/m^2^), alcohol consumption status at baseline, and prescription data for analgesic medications including opioids, antidepressants, gabapentinoids, NSAIDs, and paracetamol. To avoid reidentification of patients, any frequencies less than six were suppressed.

The outcomes considered were:

- Prevalence and incidence of opioid prescribing.
- Prevalence and incidence of other prescription medications for pain (gabapentinoids, NSAIDs, paracetamol, tricyclic antidepressants, other antidepressants).
- Mortality.

### 2.6. Statistical Analysis

Descriptive statistics were used to report outcomes using raw counts (%) and mean ± standard deviation (SD) as appropriate.

Incidence rate ratios (IRRs) were derived from Poisson regression models. Cox proportional hazards models were used to calculate adjusted hazard ratios (HRs) with 95% confidence intervals (CIs) for the time to first prescription of each medication class or mortality as appropriate. Cox proportional hazard models were adjusted for age, sex, and ethnicity. Statistical analysis was conducted using R statistical software (R Core Team, version 4.4). Statistical significance was determined by a *p*-value < 0.050.

## 3. Results

### 3.1. Study Population Characteristics

In this extract, 1771 practices were selected based on the input criteria. Of these practices, 46,599,092 patients were registered, but only 28,260,797 qualified to participate in this study based on the study period, population age, and data quality requirements. In the cohort of qualifying patients, 4967 patients with cannabis exposure were identified. Following application of exclusion criteria, 4305 cannabis-exposed patients were excluded. Of these, 4303 were excluded for having opioids prescribed prior to index date and 2 for not meeting age criteria. Therefore, 662 patients were identified in the cannabis-exposed cohort and matched to 1319 controls. The baseline characteristics of the study population are presented in Table 1.

The total follow-up time was 5485.2 person-years for the overall study, with 1408.3 person-years in the exposed cohort and 4076.9 person-years in the unexposed group.

The mean age of the overall study was 48.7 ± 10.5 years, with exposed and unexposed cohorts having mean ages of 48.6 ± 10.5 and 48.8 ± 10.5 years, respectively. Most participants were male (79.6%, *n* = 1576), with 526 (79.5%) males in the exposed group and 1050 (79.6%) males in the unexposed group. The largest age category was 50–59 years (32.2%, *n* = 637).

Regarding ethnicity, 76.3% (*n* = 1511) of participants were White. Asian ethnicity was less common in the exposed group (2.0%, *n* = 13) compared to the unexposed group (6.7%, *n* = 88). Black ethnicity was more prevalent in the exposed group (8.9%, *n* = 59) versus the unexposed group (3.0%, *n* = 39).

### 3.2. Primary Outcomes

During the follow-up period, differences were observed between the exposed and unexposed groups across all analgesic medication categories (*p* < 0.001). The incidence rate of opioid prescribing was 15,500 per 100,000 person-years in the exposed group compared to 7520 per 100,000 person-years in the unexposed group (Table 2). Cannabis-exposed individuals demonstrated a higher likelihood of being prescribed opioids compared to unexposed controls (HR: 2.06; 95% CI: 1.74–2.43; *p* < 0.001; Table 3).

Cannabis-exposed individuals were more likely to be prescribed tricyclic antidepressants (HR: 2.64; 95% CI: 2.03–3.44; *p* < 0.001) and other antidepressants (HR: 7.22; 95% CI: 5.24–9.94; *p* < 0.001). Gabapentinoids showed the second-highest association with cannabis exposure (adjusted HR: 3.31; 95% CI: 2.34–4.67; *p* < 0.001), followed by paracetamol (HR: 3.30; 95% CI: 2.43–4.48; *p* < 0.001).

During the follow-up period, 40 (6.0%) deaths occurred in the exposed group compared to 36 (2.7%) in the unexposed group. The adjusted hazard ratio for mortality was 3.81 (95% CI: 2.29–6.31; *p* < 0.001).

## 4. Discussion

This population-based retrospective cohort study represents the first examination of the relationship between recorded cannabis use and analgesic prescribing patterns in UK patients with osteoarthritis. The findings demonstrate an association between cannabis exposure and increased prescribing across all categories of analgesic medications, including opioids, gabapentinoids, NSAIDs, paracetamol, and antidepressants. These findings must be considered in the context of the limitations of current coding practices for cannabis exposure.

The primary finding of this study was that individuals with osteoarthritis who had recorded cannabis use were twice (HR: 2.06; *p* < 0.001) as likely to be prescribed opioids compared to matched controls. Participants in the exposed cohort were also more likely to be prescribed any analgesic during the study period. This finding is contrary to the hypothesis that cannabis use might be associated with reduced opioid prescribing, as suggested by some North American studies following cannabis legalisation [31]. Observational studies which have assessed the change in opioid prescriptions among pain patients have also shown very low certainty evidence that prescribing CBMPs may reduce opioid use [30]. There are some studies from North America which have failed to show that access to cannabis has reduced either prescribed or illicit opioid use itself or subsequent adverse outcomes [32,36]. This may be reflective of the limited clinical oversight of medical cannabis use in those jurisdictions studied. Illicit cannabis use in the UK during the study period (1995–2023) is likely more reflective of jurisdictions where cannabis is legally available as either a medicine or for adult use, but clinical oversight to help reduce concurrent opioid use is absent [25]. The increase in use of analgesics among those in the cannabis-exposed cohort may therefore represent individuals who have more severe pain, rather than being a consequence of cannabis consumption.

There is limited existing evidence suggesting that cannabis use in individuals with osteoarthritis or other chronic pain conditions may conversely be associated with an increase in analgesia prescribing. However, other retrospective studies suggest that cannabis-related codes recorded in an individual’s electronic health records is associated with poorer health outcomes. A study by Glickman and colleagues of inflammatory bowel disease using the TriNetX Diamond Network found that individuals with cannabis use identified through clinical coding were associated with an increased risk of opioid use, hospitalisation, and emergency department visits [37]. In both this and the present study, it must be considered that cannabis consumption may potentially be the causative factor of the association with poorer outcomes. However, several potential explanations also exist for these findings. Firstly, the association may reflect confounding by indication, where individuals with more severe or refractory pain are both more likely to seek alternative treatments, including cannabis, and to require multiple analgesic medications [38]. The baseline characteristics support this interpretation, showing higher baseline use of pain medications in the cannabis-exposed group at the index date. Consequently, the outcomes of the present study may also reflect a marker of clinical severity, rather than a direct effect of cannabis exposure. Secondly, the clinical coding of cannabis use in primary care may be selective, potentially occurring more frequently in individuals experiencing adverse effects, dependency issues, or complications related to cannabis use, particularly as many codes specifically capture cannabis-related disorders. This selection bias could result in the cannabis-exposed cohort representing a higher-risk population with greater healthcare needs rather than typical cannabis users. Finally, socioeconomic factors may contribute to both cannabis use and increased healthcare utilisation. In the present study there is residual confounding despite attempts to match each cohort. There is a higher prevalence of current smoking in the cannabis-exposed cohort, despite attempts to match each group by smoking status. This suggests potential clustering of risk behaviours, which are associated with morbidity and greater medication requirements. In particular, there is a positive association between smoking tobacco and the prevalence of osteoarthritis and musculoskeletal pain [39,40]. In addition, there is a greater proportion of black individuals in the cannabis-exposed cohort. It has been established that there are ethnic inequalities in the prevalence of multiple long-term conditions in the United Kingdom, in addition to the quality of care provided to different ethnic groups [41,42]. Further research is needed to understand how this may directly affect the outcomes of those who consume cannabis as well.

In the present study, there was an increased risk of mortality in the cannabis-exposed group (HR: 3.81; *p* < 0.001). This contrasts with a recent study using data from the UK Biobank which demonstrated no increased risk of all-cause mortality in both males and females who reported daily or near daily cannabis use [43]. In comparison to the present study, participants in the UK Biobank self-report their cannabis use at the point of enrolment, providing improved identification of true cannabis consumption. However, the risk of cardiovascular mortality was increased in females with heavy cannabis consumption in the UK Biobank study [43]. This finding is also supported by other studies which suggest there is no increased risk of overall mortality or toxicity, but there may be increased cardiovascular risk in heavy cannabis consumers, which needs further assessment to determine the cause [43,44,45]. The elevated mortality risk in the cannabis-exposed group in the present study therefore requires careful interpretation considering the potential confounding in this cohort. This association likely reflects the complex interplay of factors associated with individuals whose cannabis use is recorded in medical records rather than a direct causal effect of cannabis. Potential contributing factors to this result include higher rates of mental health conditions, substance use disorders, socioeconomic disadvantage, and comorbid medical conditions. In some cases, those potential confounders may be a reason behind an individual’s cannabis use [14], particularly as studies where cannabis use is assessed through self-reporting do not find the same outcomes [43,44,45].

This study has several important limitations that must be considered when interpreting the results. Firstly, the reliance on clinical coding for cannabis exposure likely results in misidentification of cannabis use status. The latest survey data from the Office for National Statistics suggests that 7.6% of people aged 16 to 59 years reported cannabis use in the 12 months preceding March 2023 [46]. Analysis of a nationally representative cross-sectional survey conducted by our group also estimates that 6.4% of individuals with a diagnosed health condition and 11.4% of people with chronic pain report using illegal cannabis to self-treat their condition [14]. Finally, there are a growing number of individuals who are prescribed CBMPs since legalisation in November 2018 [26,47]. This may not be reliably captured in UK primary care as these are initiated by specialist physicians, and fewer than 5 individuals have accessed unlicensed CBMPs through the National Health Service [48]. This study therefore captures only a small subset of cannabis users whose use was documented in primary care. This cohort introduces a selection bias whereby individuals experiencing the negative sequalae of cannabis use, rather than the broader and largely undocumented population of self-medicating patients. Secondly, the temporal relationship between cannabis initiation and analgesic prescribing cannot be definitively established. Due to the lack of clinical coding of cannabis exposure, the inclusion criteria in the cannabis-exposed cohort extended to any code before or after the index date. This assumption was made on the basis that cannabis consumption is likely long-term in those with any relevant clinical code. However, some patients may have started using cannabis after experiencing inadequate pain relief from prescribed medications, while others may have started consuming cannabis prior to the development of osteoarthritis. Moreover, the study lacks information about cannabis products, dosing, frequency of use, or duration of exposure. Cannabis encompasses a wide variety of products with different cannabinoid profiles and routes of administration, each potentially having different effects on pain and healthcare utilisation. Moreover, illegal cannabis is at risk of adulteration and contamination, which may expose individuals to potentially harmful moulds, bacteria, and scheduled drugs [49,50,51,52]. In addition, unmeasured confounding remains a significant concern. Despite matching on several variables and adjusting for demographic factors, important confounders such as pain severity, functional status, mental health conditions, other substance use, and socioeconomic status were not fully captured in the analysis. Complementary to this, despite attempts to match by smoking status, due to the high prevalence of current tobacco consumption in the cannabis-exposed cohort, there was a much higher proportion of individuals who were current smokers in this group. These could each have an impact on the severity of osteoarthritis, likelihood of consuming cannabis, and other health outcomes. This limits the ability to infer the direct effects of cannabis beyond these on the outcomes presented within the analysis. Finally, the generalisability of findings to other healthcare systems or populations may be limited, given the specific context of UK primary care and the legal status of cannabis during the study period.

The study results emphasise the need for high-quality health technology assessment examining the efficacy and safety of treating osteoarthritis-associated chronic pain with CBMPs. The two limited trials conducted to date are insufficient to inform clinical practice on a population basis [23,24], leaving patients to seek alternatives through unregulated channels. Research examining the impact of regulated medical cannabis programmes on prescribing patterns and patient outcomes would also be particularly valuable. Such studies could help determine whether legal access to standardised cannabis products affects conventional medication use and healthcare utilisation differently than illegal products. The study also highlights the importance of accurate clinical coding for conducting population-level research and informing individual level care [53]. Considering the estimated prevalence of cannabis use in the general population [14,46], electronic healthcare record companies should strongly consider including codes for collection of data on cannabis use in primary care that is comparable to tobacco and alcohol. Finally, permitting accurate recording of CBMP prescribing into primary care records could improve understanding of the effects of this class of medications through observational methodologies.

## 5. Conclusions

This retrospective cohort study demonstrates an association between recorded cannabis exposure and increased prescribing of analgesic medications among UK patients with osteoarthritis, though the findings are likely influenced by coding limitations, residual confounding, and selection bias. The study highlights the limitations of current clinical coding practices for cannabis use. Among the small population of cannabis consumers identified, the relationships observed may be confounded by socioeconomic status and a higher likelihood of coding cannabis exposure in those already experiencing adverse effects or cannabis misuse disorder. Within these limitations, the results likely reflect the complex health and social challenges faced by this population rather than direct causal effects of cannabis. This underscores the importance of improved coding practices for recording cannabis consumption in primary care and for high-quality health technology assessments to establish the efficacy and safety of CBMPs for chronic pain.

## Figures and Tables

**Table 1:** Baseline Characteristics of Study Population.

Covariate	Overall (*n* = 1981)	Unexposed (*n* = 1319)	Exposed (*n* = 662)
Age; years (mean ± SD)	48.7 ± 10.5	48.8 ± 10.5	48.6 ± 10.5
Age Categories; years (%)			
20–29	63 (3.2%)	42 (3.2%)	21 (3.2%)
30–39	384 (19.4%)	254 (19.3%)	130 (19.6%)
40–49	603 (30.4%)	403 (30.6%)	200 (30.2%)
50–59	637 (32.2%)	427 (32.4%)	210 (31.7%)
60–69	262 (13.2%)	170 (12.9%)	92 (13.9%)
70–79	23 (1.2%)	17 (1.3%)	6 (0.9%)
80+	<10 (0.5%)	<10 (0.5%)	<6 (0.5%)
Sex (%)			
Male	1576 (79.6%)	1050 (79.6%)	526 (79.5%)
Female	405 (20.4%)	269 (20.4%)	136 (20.5%)
Ethnicity (%)			
White	1511 (76.3%)	1000 (75.8%)	511 (77.2%)
Asian	101 (5.1%)	88 (6.7%)	13 (2.0%)
Black	98 (4.9%)	39 (3.0%)	59 (8.9%)
Mixed	<15 (0.5%)	<10 (0.5%)	<6 (0.5%)
Other	25 (1.3%)	14 (1.1%)	11 (1.7%)
Missing	236 (11.9%)	171 (13.0%)	65 (9.8%)
BMI; kg/m^2^ (mean ± SD)	27.2 ± 5.35	27.5 ± 5.12	26.9 ± 5.75
Current Alcohol Consumer (%)	1606 (81.1%)	1038 (78.7%)	568 (85.8%)
Current Smoker (%)	1121 (56.6%)	576 (43.7%)	545 (82.3%)
Medications (%)			
Paracetamol	209 (10.6%)	96 (7.3%)	113 (17.1%)
NSAIDs	1285 (64.9%)	860 (65.2%)	425 (64.2%)
Gabapentinoids	74 (3.7%)	36 (2.7%)	38 (5.7%)
Tricyclic Antidepressants	282 (14.2%)	152 (11.5%)	130 (19.6%)
Other Antidepressants	180 (9.1%)	49 (3.7%)	131 (19.8%)

BMI—body mass index; NSAIDs—non-steroidal anti-inflammatory drugs; SD—standard deviation. To avoid reidentification of patients, all frequencies less than six are suppressed.

**Table 2:** Incidence Rates and Unadjusted Incidence Rate Ratios for Analgesic Prescribing Outcomes and Mortality.

Outcome	Exposed Events (*n* = 662)	Unexposed Events (*n* = 1319)	Exposed IR per 100,000 Person-Years	Unexposed IR per 100,000 Person-Years	Unadjusted IRR (95% CI)	*p*-Value
Opioids	248 (37.5%)	340 (25.8%)	15,500	7520	2.06 (1.75–2.42)	<0.001
Gabapentinoids	72 (10.9%)	65 (4.9%)	5190	1560	3.33 (2.38–4.66)	<0.001
NSAIDs	319 (48.2%)	445 (33.7%)	23,700	10,900	2.18 (1.89–2.52)	<0.001
Other Antidepressants	140 (21.1%)	54 (4.1%)	9660	1340	7.21 (5.26–9.86)	<0.001
Paracetamol	96 (14.5%)	89 (6.7%)	6810	2140	3.18 (2.38–4.24)	<0.001
Tricyclic Antidepressants	117 (17.7%)	127 (9.6%)	8070	2960	2.72 (2.12–3.50)	<0.001
Mortality	40 (6.0%)	36 (2.7%)	2840	883	3.22 (2.05–5.05)	<0.001

CI—confidence interval; IR—incidence rate; IRR—incidence rate ratio; NSAIDs—non-steroidal anti-inflammatory drugs.

**Table 3:** Adjusted Hazard Ratios for Analgesic Prescribing Outcomes and Mortality.

Outcome	Exposed Events (*n* = 662)	Unexposed Events (*n* = 1319)	Adjusted HR (95% CI)	*p*-Value
Opioids	248 (37.5%)	340 (25.8%)	2.06 (1.74–2.43)	<0.001
Gabapentinoids	72 (10.9%)	65 (4.9%)	3.31 (2.34–4.67)	<0.001
NSAIDs	319 (48.2%)	445 (33.7%)	1.99 (1.72–2.31)	<0.001
Other Antidepressants	140 (21.1%)	54 (4.1%)	7.22 (5.24–9.94)	<0.001
Paracetamol	96 (14.5%)	89 (6.7%)	3.30 (2.43–4.48)	<0.001
Tricyclic Antidepressants	117 (17.7%)	127 (9.6%)	2.64 (2.03–3.44)	<0.001
Mortality	40 (6.0%)	36 (2.7%)	3.81 (2.29–6.31)	<0.001

CI—confidence interval; HR—hazard ratio; NSAIDs—non-steroidal anti-inflammatory drugs.

## Data Availability

Access to Clinical Practice Research Datalink data is subject to protocol approval via the Clinical Practice Research Datalink Research Data Governance Process.

## Supplementary Materials

The following supporting information can be downloaded at: https://www.mdpi.com/article/10.3390/medicines12040027/s1. Supplementary Data File S1: Osteoarthritis_MM_birm_cam_CPRD_AURUM.xlsx. Supplementary Data File S2: Cannabis Exposure Medical Dictionary.xlsx.

## Abbreviations

The following abbreviations are used in this manuscript:

BMI	Body mass index
CB1	Cannabinoid receptor 1
CB2	Cannabinoid receptor 2
CBD	Cannabidiol
CBMPs	Cannabis-based medicinal products
CI	Confidence Interval
CPRD	Clinical Practice Research Datalink
DExtER	Data Extraction for Epidemiological Research
HR	Hazard Ratio
IRR	Incidence Rate Ratio
NSAIDs	Non-steroidal anti-inflammatory drugs
RCTs	Randomised controlled trials
SD	Standard Deviation
THC	(-)-trans-Δ9-tetrahydrocannabinol
TRPV1	Transient receptor potential vanilloid 1
